# Seroprevalence and Molecular Identification of *Brucella* spp. in Camels in Egypt

**DOI:** 10.3390/microorganisms8071035

**Published:** 2020-07-13

**Authors:** Aman Ullah Khan, Ashraf E. Sayour, Falk Melzer, Sherif Abdel Ghafar Elsayed El-Soally, Mandy C. Elschner, Waleed S. Shell, Amira A. Moawad, Shereen Aziz Mohamed, Ashraf Hendam, Uwe Roesler, Heinrich Neubauer, Hosny El-Adawy

**Affiliations:** 1 Friedrich-Loeffler-Institut, Institute of Bacterial Infections and Zoonoses, 07743 Jena, Germany; AmanUllah.Khan@fli.de (A.U.K.); falk.melzer@fli.de (F.M.); mandy.elschner@fli.de (M.C.E.); amira.moawad@fli.de (A.A.M.); Heinrich.neubauer@fli.de (H.N.); 2 Institut for Animal Hygiene and Environmental Health, Free University of Berlin, 14163 Berlin, Germany; uwe.roesler@fu-berlin.de; 3 Department of Pathobiology, College of Veterinary and Animal Sciences, Jhang 35200, Pakistan; 4 Department of Brucellosis, Animal Health Research Institute, Agricultural Research Center, Dokki 12618, Giza, Egypt; sayourashraf@gmail.com; 5 Veterinary Service Department, Armed Forces Logistics Authority, Egyptian Armed Forces, Nasr City 11765, Egypt; dr.sherifelsoaaly@gmail.com; 6 Central Laboratory for Evaluation of Veterinary Biologics, Agricultural Research Center, Abbasaia 11517, Cairo, Egypt; tarikwaleedshell@hotmail.com; 7 Provincial Laboratory, Institute of Animal Health Research, Agricultural Research Center, Mansoura 35516, Egypt; 8 Veterinary Serum and Vaccine Research Institute, Agricultural Research Center, Abbasaia 11517, Cairo, Egypt; shereenibraheem1968@gmail.com; 9 Climate Change Information Center, Renewable Energy and Expert Systems (CCICREES), Agricultural Research Center, 9 Algamaa Street, Giza 12619, Egypt; a_hendam@hotmail.com; 10 Faculty of Veterinary Medicine, Kafrelsheikh University, Kafr El-Sheikh 33516, Egypt

**Keywords:** brucellosis, camel, *B. suis*, Egypt, seroprevalence, real-time PCR

## Abstract

Brucellosis is one of the most important worldwide zoonoses of many countries including Egypt. Camel brucellosis has not gained much attention in Egypt yet. This study is focused on the three governorates with the highest camel populations and the largest camel markets in the country to determine the disease seroprevalence and identify the *Brucella* species in local camel holdings. In total, 381 serum samples were collected from male and female camels from Giza, Aswan, and Al-Bahr Al-Ahmar (the Red Sea) governorates. Samples were serologically examined using the Rose–Bengal plate test (RBPT), indirect ELISA (i-ELISA), competitive ELISA (c-ELISA) and complement fixation test (CFT). *Brucella* antibodies were detected in 59 (15.5%), 87 (22.8%), 77 (20.2%) and 118 (31.0%) of sera by RBPT, i-ELISA, c-ELISA and CFT, respectively. Using real-time PCR, *Brucella* DNA was amplified in 32 (8.4%) seropositive samples including *Brucella abortus* (25/32), *Brucella suis* (5/32) and *Brucella melitensis* (2/32), defining a complex epidemiological status. To the best of our knowledge, this is the first study reporting *Brucella suis* DNA in camel serum. The risk-associated factors including age, sex, breed and geographical distribution were statistically analyzed, showing non-significant association with seroprevalence. The results of this study will raise awareness for camel brucellosis and help develop effective control strategies.

## 1. Introduction

Brucellosis is a global zoonotic disease affecting cattle, sheep, goats, camels, pigs and wildlife as well as humans. It is well controlled in many countries but is still endemic in many others with high records in humans in the Middle East and central Asian regions [1]. Brucellosis in camels was first reported in 1931 [2]. Since then, it has been testified by all camel rearing countries like Sudan, Ethiopia, Somalia, Kenya, Nigeria, Jordan and Egypt but not Australia [3]. There is no separate specific species of *Brucella* that displays a preference for camels as they can be infected by those that have already been shown to be prevalent in bovines, ovines and caprines [4,5,6]. The clinical picture of brucellosis in camels can vary from asymptomatic to abortion, retention of fetal membranes, weak offspring, impaired fertility and delayed sexual maturity in females and orchitis accompanied by lameness in males [2,4,7].

The 120000 camels kept in Egypt represent 1.1% and 0.9% of the total number of camels in Arab countries and Africa, respectively [8]. Higher numbers of camels are raised in countries of the Horn of Africa (Djibouti, Eritrea, Somalia and Ethiopia) as well as parts of Kenya, Sudan, and Uganda [2]. Camels are usually imported from Sudan to Egypt. About half of the camel population lives in the Shalateen area of Al-Bahr Al-Ahmar (the Red Sea) governorate.

Brucellosis has been endemic in Egypt for thousands of years [9,10]. The disease has been detected in livestock predominantly in ruminants with prevalences from 2.47% to 26.66% [11,12,13]. *Brucella abortus* and *B. melitensis* were isolated from all livestock species and humans but *B. suis* was identified in cattle and pigs only [10,14,15]. There are few seroprevalence reports on camel brucellosis in Egypt, as the disease has not received much attention.

For serological testing, a screening test of high sensitivity is usually followed by a confirmatory test of high specificity [16]. Rose-Bengal plate test (RBPT), complement fixation test (CFT), standard agglutination test (SAT), competitive enzyme-linked immunosorbent assay (c-ELISA), fluorescence polarization assay (FPA) and indirect ELISA (i-ELISA) have been used for detection of anti-*Brucella* antibodies in camel sera [2]. Culture of brucellae is sometimes difficult and time consuming. Additionally, this method poses a risk to laboratory personnel and requires specific biosafety measures [17]. Thus, detection of *Brucella* DNA by PCR in clinical samples is considered a preferred tool for definitive diagnosis of brucellosis [18]. Combination of PCR with at least one of the conventionally used serological tests (e.g., RBPT, SAT, ELISA) was recommended for developing countries [19].

Infection in humans may occur by direct contact with infected animals or consumption of contaminated raw camel milk [2]. Outbreaks of human brucellosis by consumption of infected raw camel milk have been reported in Qatar, Israel and countries of the African Horn [20,21,22]. Brucellosis proved to be a serious occupational health hazard to livestock handlers especially abattoir workers in Egypt [23].

Considering public health concerns and zoonotic importance of brucellosis, the present study aimed at serological monitoring of camelid brucellosis with molecular identification of *Brucella* species involved in Egypt, filling a gap in knowledge of the disease epidemiology.

## 2. Materials and Methods

### 2.1. Study Area and Sera Collection

**Figure 1:**
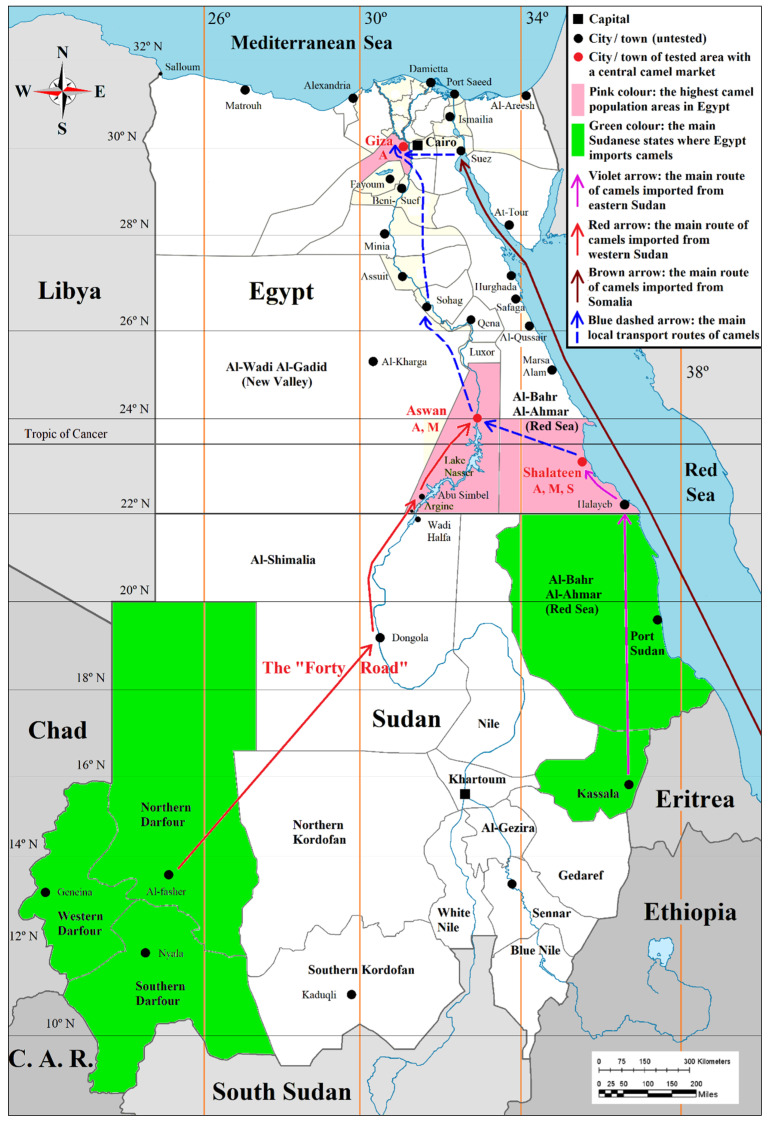
Map of Egypt showing the geographic distribution and the main portals of entry of camels imported from Sudan and Somalia, as well as the main local routes of camel transport to central markets.

The study was conducted from March 2017 to November 2019. Sera of the Arabian one-humped or dromedary camel (*Camelus dromedarius*) were collected from Giza, Aswan and Al-Bahr Al-Ahmar (the Red Sea) governorates in Egypt. These governorates house the highest camel populations and the largest camel markets, *viz*. Birqash market in Imbaba (Giza), Daraw market in Aswan and Shalateen International Market in Al-Bahr Al-Ahmar (the Red Sea). The main portals of entry for camels imported from Sudan and Somalia, as well as the main local routes of camel transport to central markets, are shown in Figure 1. Camels usually live for some years on farms to produce milk, to be used for cheesemaking, tourism, etc.

The data for each sample including origin, sex, breed and age were recorded. In total, 381 serum samples (106 from Giza, 186 from Aswan, and 89 from Al-Bahr Al-Ahmar (the Red Sea)) from domestic camels were collected in sterile vacutainer tubes without anticoagulant. The serum was separated and stored at −20 °C.

### 2.2. Ethics Statement

This study was carried out in strict accordance with the Guidelines of the Egyptian Network of Research Ethics Committees (ENREC), which complies with the international laws and regulations regarding the ethical considerations in research. All efforts were made to minimize animal suffering and to reduce the number of animals used.

### 2.3. Detection of Anti-Brucella Antibodies

All sera were screened for anti-*Brucella* antibodies by RBPT (IDEXX, Westbrook, ME, USA), indirect ELISA (ID Screen^®^ Brucellosis Serum Indirect Multi-species (protein G-HRP conjugate), IDVet Innovative Diagnostics, Grabels, France), c-ELISA (SVANOVIR^®^ *Brucella*-Ab c-ELISA kit, Boehringer Ingelheim Animal Health International GmbH, Ingelheim, Germany) and complement fixation test (CFT) according to the manufacturers‘ instructions.

These tests are mainly standardized for use in cattle, but the OIE recommends their use in camels as well after validation [24]. The RBPT antigen was standardized against the OIE International Standard Serum (OIEISS) to give a positive reaction at a dilution of 1:45 and a negative reaction at a dilution of 1:55. The CFT followed the range of recommendations by the OIE [24]. This included an antigen standardized to give 2:200 of the OIEISS (one 50% hemolytic unit), 2% sheep RBCs, two full (100% hemolysis) units of complement and four full units of hemolysin. Serum showing a value ≥ 20 ICFTU/mL of the OIEISS was considered positive for CFT. ELISA methods conducted and results were calculated in accordance with the manufacturer’s instructions.

### 2.4. Molecular Detection of Brucella spp. DNA

DNA was extracted automatically from serum samples by QIAcube machine (QIAGEN, Hilden, Germany) using the QIAamp DNA Mini Kit (QIAGEN, Hilden, Germany) according to the instructions of the manufacturer. Reference strains of *Escherichia coli* (ATCC 25922) and serum form non infected animals were used as negative extraction control in each cycle. Genus *Brucella* and species-specific (*B. abortus*, *B. melitensis* and *B. suis*) real-time PCRs were used for detection of *Brucella* DNA. PCR was performed using primer and probe (Jena Bioscience GmbH, Jena, Germany) sets as given in Table 1.

The PCR protocol was modified (volume and temperature) than previously published [25,26] to obtain optimal results as DNA used in this study as template was extracted from serum not from bacterial colonies. Briefly, PCR reaction was performed in 15 μL multiplex PCR mixture with 2× TaqMan™ environmental master mix (Applied Biosystems^®^, Darmstadt, Germany), 0.2 µM of each primer, 0.1 µM of each probe and 5 μL of template DNA. Amplification and real-time fluorescence detection were carried out on a CFX96™ Real-Time PCR Detection System (Bio-Rad Laboratories, Inc., Hercules, CA, USA). The reaction conditions were: decontamination at 50 °C for 2 min, initial denaturation at 95 °C for 10 min followed by 50 cycles of denaturing at 95 °C for 25 s and anealing/elongation at 57 °C (*B. abortus* and *B. melitensis*) [25] and 60 °C (*B. suis*) [26] for one minute. Sample data scores were confirmed by visual inspection of graphical plots and Cycle Threshold (CT) values for each sample were obtained. CT values ≤ 38 were considered positive after in house validation to avoid false positive results. The CT values of negative extraction controls were either > 38 or not detected. Reference strains of *B. abortus* S-99 (ATCC 23448), *B. melitensis* 16M (ATCC 23456) and *B. suis* biovar 1 (ATCC 23444) were used as positive controls. Reference strains of *Escherichia coli* (ATCC 25922), *Staphylococcus aureus* (ATCC 25923) and *Ochrobactrum intermedium* (DSM 17986) were used as negative controls.

### 2.5. Statistical Analysis

The agreement of positive camel results of serological tests and real-time PCR was expressed using Venn diagrams. Correlation of potential risk factors (geographical location, breed type, sex and age) with seroprevalence and molecular detection of 381 camels was analyzed using Pearson’s Chi-squared test (X^2^) and odds ratio (OR). The estimation of X^2^ was done using RStudio Version 1.1.463.

## 3. Results

### 3.1. Seroprevalence of Anti-Brucella Antibodies in Camel Sera

Out of 381 camel serum samples, 59 (15.5%), 87 (22.8%), 77 (20.2%) and 118 (31.0%) were found positive for *Brucella* antibodies by RBPT, i-ELISA, c-ELISA and CFT, respectively (Table 2). Higher numbers of seropositive animals, i.e., 17.7%, 25.8%, 22.0% and 31.7% were detected from Aswan governorate using RBPT, i-ELISA, c-ELISA and CFT, respectively. The corresponding values from Giza governorate were 14.2%, 22.6%, 14.2% and 31.1% and from Al-Bahr Al-Ahmar (the Red Sea) governorate were 12.4%, 16.9%, 23.6% and 29.2% in that order. Only 16 serum samples were found seropositive by all serological tests.

Higher seroprevalences were recorded in male animals (17.6%, 24.1%, 23.4% and 34.9%) than female animals (8.1%, 18.6%, 12.8% and 17.4%) using RBPT, i-ELISA, c-ELISA and CFT, respectively, in the investigated governorates (Table 2).

Correlation of potential risk factors (geographical location, breed, sex and age) with seroprevalence and molecular detection of *Brucella* spp. in 381 camels is shown in Table 3. Using i-ELISA, 25.8% of seropositive samples were found in Aswan, 22.6% in Giza and 16.9% in Al-Bahr Al-Ahmar (the Red Sea) (Table 3).

The seroprevalence in different breeds using i-ELISA was 16.9% (15 out of 89) in Al-Beshary, 28.0% (26 out of 93) in Al-Ebadi, 22.6% (24 out of 106) in Al-Zemkly and 23.7% (22 out of 93) in Al-Zubaidi breeds (Table 3).

The results of this study showed relatively higher seropositive males (17.6%, 24.1% and 23.4%) than females (8.1%, 18.6% and 12.8%) using RBPT, iELISA and cELISA, respectively with confidence intervals (95% CI) 0.2819–2.2582, 0.6333–3.1044 and 0.4215–2.4729, respectively.

Seroprevalences of age groups were 22.9% (52/227), 27.9% (19/68), 13.7% (7/51) and 25.7% (9/35) in animals of <8 years, ≥8–11 years, 11–13 years and >13–15 years using i-ELISA, respectively. In the univariate analysis based on i-ELISA, all variables (geographical location, breed, age and sex) showed no association with seroprevalence (Table 3).

### 3.2. Detection of Brucella spp. DNA in Camel Sera

*Brucella* DNA was detected in serum samples positive by either RBPT, i-ELISA, c-ELISA or CFT. *Brucella* DNA was detected in 32 (8.4%) samples and was typed as *B. abortus* (25/32), *B. suis* (5/32) and *B. melitensis* (2/32) (Table 2). *Brucella* DNA was detected in 4.3%, 8.49% and 16.8% of camels from Aswan, Giza and Al-Bahr Al-Ahmar (the Red Sea), respectively. *Brucella* DNA was amplified in 8.14% (24 out of 295; 17 *B. abortus*, 5 *B. suis* and 2 *B. melitensis*) and 9.3% (8 out of 86; *B. abortus*) samples of male and female camels, respectively. The DNA concentration of positive samples was not sufficient for optimal sequencing. Sera from Al-Bahr Al-Ahmar (the Red Sea) governorate were more often positive in PCR (16.8%) than those of Giza (8.5%) and Aswan governorates (4.3%).

*Brucella* DNA was identified in all camel breeds (Table 3). Identification of *B. suis* DNA from camel sera is a new finding of this study. *B. suis* was only identified in 5 seropositive male animals of breed Al-Beshary in Al-Bahr Al-Ahmar (the Red Sea) governorate. The *Brucella* DNA extracted from seronegative samples by either RBPT, i-ELISA, c-ELISA or CFT was either not amplified or showed CT values > 38 by real-time PCR.

### 3.3. Statistical Analysis

**Figure 2:**
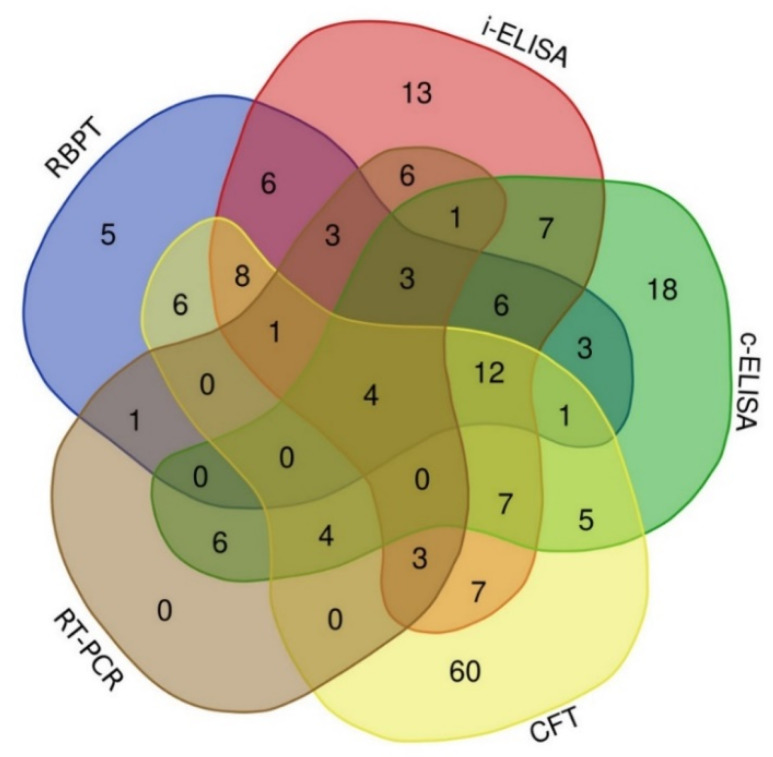
Venn diagram showing the agreement of positive results of serological tests and real-time PCR.

Investigating the agreement of the results of positive cases detected by serological tests and real-time PCR, the Venn diagram (Figure 2) reveals that 60, 18, 13 and 5 camels were identified as positives by CFT, c-ELISA, i-ELISA and RBPT only. There were only four animals classified as positive by all serological assays and real-time PCR. The CFT agreed with real-time PCR, i-ELISA, c-ELISA and RBPT in 12, 34, 33 and 32 positive animals, respectively. Indirect ELISA and c-ELISA had 40 positive results (Figure 2).

## 4. Discussion

Despite the available data on *Brucella* infection in humans and ruminants, little is known about the status of camel brucellosis in Egypt. The identification of *Brucella* spp. in various farm animals and wildlife species (*viz*. cattle, buffalo, sheep, goat, camel, bison, African buffalo, Alpine ibex) highlights their role in disease spread [27,28,29,30,31]. Consumption of raw milk and dairy products of infected camels was associated with brucellosis in humans [20,21,22]. This study is investigating camel brucellosis in three Egyptian governorates with the highest number of camels, *viz*. Giza, Aswan and Al-Bahr Al-Ahmar (the Red Sea). The latter two governorates are the main entry portals for camels imported from Sudan. Giza receives imported Sudanese camels from Aswan as well as Somali camels shipped to the port of Suez. Apart from camels smuggled through the desert, Egypt has been importing camels officially from east Africa where brucellosis is enzootic in ruminants including camels [19,32,33]. The fact that these camels are imported does not preclude the possibility of acquiring brucellosis from a local source.

In this study, 381 camel sera were investigated serologically and 59 (15.5%), 87 (22.8%), 77 (20.2%) and 118 (31.0%) were found positive for anti-*Brucella* antibodies by RBPT, i-ELISA, c-ELISA and CFT, respectively (Table 2). A previous report from camel-keeping countries has revealed seroprevalence of camel brucellosis ranging from 1.0 to 23.3% [4]. The seroprevalence figures of brucellosis in apparently healthy Sudanese camels were 79.3%, 71.4%, 70.7%, 70.6% and 68.8% using FPA, CFT, RBT, SAT and c-ELISA [19]. In camels, the prevalence was 12.9% in the Shalateen region of the Red Sea governorate [5]. One study from the regions of Siwa Oasis, Asyut and Cairo reported a prevalence of 4.17% using RBPT [34]. A similar study from Beheira district revealed prevalence figures of 8.74% and 9.26% using RBPT and ELISA, respectively [35]. The general consistency of seroprevalence data from all the governorates as revealed by i-ELISA (25.8%, 22.6% and 16.9%) in Aswan, Giza and Al-Bahr Al-Ahmar respectively) is a reflection of the continuous flow and regular distribution of imported camels from the same source countries.

In the current study, *Brucella* DNA was detected in 32 (8.4%) of all investigated camel sera (Table 2). Quantitative multiplex real-time PCR confirmed the presence of *Brucella* DNAs of 25 *B. abortus*, 5 *B. suis* and 2 *B. melitensis*. Detection of *B. abortus* DNA in the three target governorates in addition to *B. melitensis* in camels reared in Aswan and Al-Bahr Al-Ahmar (the Red Sea) governorates was expected as previous reports showed the endemicity of *B. melitensis* and *B. abortus* in these regions already [10]. Previously, *B. melitensis* was isolated from camel stomach contents of an aborted fetus [5] as well as from whole citrated blood samples from Al-Bahr Al-Ahmar (the Red Sea) governorate [36]. Similar studies reported the identification of *B. melitensis* DNA from camel milk from Giza and Aswan [34,37]. The source of *B. melitensis* in camels might be attributed to small ruminants as camels are usually reared in herds with sheep and goats in mobile flocks [31,38].

As *B. abortus* is enzootic in Egypt, the detection of a relevant number of camel sera containing *B. abortus* DNA might indicate that *B. abortus* may be the predominant spp. in camels in this region but more results are needed to confirm this. *Brucella abortus* has been isolated from camels in Sudan and it can be speculated that camels were infected by cattle, the primary hosts of *B. abortus* [39]. These data do not allow to speculate if camels were already infected when imported or that they got infected in Egypt. Interestingly, more positive serum samples were collected from Al-Bahr Al-Ahmar (the Red Sea) governorate sharing common borders with Sudan. This governorate hosts about half of the Egyptian camels, a fact that may favour the spread of brucellosis in these regions [8]. The very low amount of *Brucella* DNA extracted from camel sera hindered biotyping. Further investigation of the Egyptian *B. abortus* strain is necessary to prove or deny the epidemiological relation with the Sudanese *B. abortus* (biovar 6) strains detected in cattle in Darfour [40] and sheep in Kassala [41] as well as *B. abortus* biovar 3 from camels in Eastern Sudan [42]. Camel herds move between the states of North Sudan [43] and they are reared with cattle, sheep and goats [31]. Camels from the whole Darfour sector usually gather at Southern Darfour during the autumn months seeking water of the tropical heavy rain season. There, they also mingle intensely with cattle again. Unlike camels, Sudanese cattle do cross the Sudanese borders during the dry season to South Sudan, Central African Republic and Congo reaching as far as Niger and Mali to the west.

The detection of *B. suis* DNA is a new finding of this study probably attributable to the first use of recently developed highly sensitive and specific primer for *B. suis* biovars 1 to 4 to test camel sera [26]. However, the identification of *B. suis* in the present study is not unexpected as *B. suis* has previously been isolated from cattle [10] and *B. suis* or its DNA was identified in pigs in Egypt [15,44].

The source of *B. suis* in camels could be traceable to either domestic or wild pigs, e.g., the wild boars (*Sus scrofa*) of the adjacent Eastern Desert. Being a border governorate with Sudan, Al-Bahr Al-Ahmar (the Red Sea), is also likely to have *B. suis* imported from Sudan, where some pig farms in Khartoum state to the west of Kassala state exist. The uncontrolled transboundary movement of Sudanese cattle to adjacent African countries, i.e., South Sudan and Central African Republic may contribute to the spread of *B. suis* as both states have domestic and wild pigs [45]. The countries of the Horn of Africa, with huge camel populations have similar restricted pig populations comparable to Egypt in contrast to Uganda [45]. The exact source of *B. suis* should be traced to stop further transmission as camels could have acquired the disease from a local source.

Identification of risk factors is crucial for control of brucellosis. Animal related risk factors (age, sex, breed and species), farm management, geographical distribution, herd management and farmers‘ awareness of brucellosis have been associated with the prevalence of brucellosis [46]. In this study, the seroprevalences of age groups were 22.9% (52/227), 27.9% (19/68), 13.7% (7/51) and 25.7% (9/35) in animals of <8 years, ≥8–11 years, 11–13 years and >13–15 years using i-ELISA, respectively. In a previous study, the seroprevalence was significantly higher (29.4%) in camels brought for slaughtering at Akaki abattoir, Ethiopia of the 5–9 years age group when compared to other age groups (0–4.8%) using RBPT [47]. However, known risk factors (age, sex, breed and locality) were found unrelated, which is consistent with a previously published report [48]. Rearing of camels with other farm animals might be identified as an important risk factor of camel brucellosis as previously described [2,47,49,50].

None of the tests can differentiate among *B. abortus*, *B. suis* and *B. melitensis*. Many immunoassays are available with different sensitivity and specificity but they must be used in accordance with strict standardization rules and meet the requirements laid down by the OIE [24]. An obvious discrepancy among the tests used in this study was seen: (CFT (31.0%), i-ELISA (22.8%), c-ELISA (20.2%), RBPT (15.5%) and real-time PCR (8.4%)). Although these samples were not taken according to the sampling plan of the Egyptian surveillance policy of ruminants, the ranges were in agreement with the previously published reviews and reports of camel brucellosis (ranging from 1.0% to 24.0%) in Egypt [4,5,34,51].

Interestingly, the results of this study showed relatively higher seropositive males (17.6%, 24.1% and 23.4%) than females (8.1%, 18.6% and 12.8%) using RBPT, i-ELISA and c-ELISA, respectively. This may be due to the fact that the vast majority of imported camels are males for slaughter with some females that farmers usually keep for breeding.

Of the 32 real-time PCR positive cases, the i-ELISA identified 21 followed by the c-ELISA (18) and the CFT and the RBPT each detected 12. In terms of positive camel recognition, the CFT revealed the highest number (118), followed by the i-ELISA (87), the c-ELISA (77), the RBPT (59), and finally the real-time PCR (32), with exclusive detections of 60, 18, 13, 5 and 0 by every single test, respectively. It is noteworthy that the 60 CFT positive samples that were negative by all other tests revealed low titers of 1:10 or 1:20 and rarely 1:40.

The nature of camelid humoral immune response and the unique nature of their heavy chain antibodies might be an explanation for these puzzling results. A reduced sensitivity of c-ELISA has been reported previously [52]. These findings call for validation and standardization of tested kits for camel brucellosis and in the worst case scenario for the development of new “camelid” diagnostics.

## 5. Conclusions

Under conditions of this investigation, DNA of three *Brucella* species was identified in 32 camel sera. *B. abortus* was the most common (25 camels), followed by *B. suis* in 5 camels and *B. melitensis* in only 2 camels. To the best of our knowledge, this is the first study reporting the identification of *B. suis* DNA in serum from camels. As camels in this study were apparently healthy, we believe that camels can act both as a reservoir of brucellosis and as a source of infection to other camels. The relative high seropositive camels in this study might reflect that the camels were imported from brucellosis infected herds.

The endemic nature of the disease together with the DNA identification of the three classic *Brucella* species in camel sera demonstrate complicated epidemiological situation that needs careful handling. Further investigation is needed to assess the prevalence of *Brucella* species particularly *B. suis* in camels as well as biovar and genotype identification. More attention should be paid to the standardization of serological tests for brucellosis diagnosis in camels.

## Figures and Tables

**Table 1:** Primer and probe sequences used in real-time PCR assays for the detection of *Brucella* spp., *B. abortus*, *B. melitensis* and *B. suis* in camel sera, Egypt.

Target	Primer and Probe Sequences		Reference
*Brucella* spp.	5′-GCT CGG TTG CCA ATA TCA ATG C-3´	Forward	[25]
	5′-GGG TAA AGC GTC GCC AGA AG-3´	Reverse	
	6-FAM-AAA TCT TCC ACC TTG CCC TTG CCA TCA-MGB	Probe	
*B. abortus*	5′-GCG GCT TTT CTA CGG TAT TC-3´	Forward	
	5′-CAT GCG CTA TGA TCT GGT TAC G-3´	Reverse	
	Hex-CGC TCA TGC TCG CCA GAC TTC AAT G-BHQ1	Probe	
*B. melitensis*	5′-AAC AAG CGG CAC CCC TAA AA-3´	Forward	
	5′-CAT GCG CTA TGA TCT GGT TAC G-3´	Reverse	
	Cy5-CAG GAG TGT TTC GGC TCA GAA TAA TCC ACA-BHQ2	Probe	
*B. suis*	5′-GCC AAA TAT CCA TGC GGG AAG-3´	Forward	[26]
	5′-TGG GCA TTC TCT ACG GTG TG-3´	Reverse	
	VIC-TTG CGC TTT TGT GAT CTT TGC TTA TGG-MGB	Probe	

**Table 2:** Seroprevalence and molecular identification of *Brucella* DNA in camel sera collected from Giza, Aswan and Al-Bahr Al-Ahmar (the Red Sea) governorates, Egypt.

Governorate	Sex	Number of Samples	Seroprevalence *n* (%)				Molecular Identification		
			RBPT *	i-ELISA *	c-ELISA *	CFT *	Real-Time-PCR n (%)	*Brucella* spp. DNA Identification	Cq/Ct-Values **
Giza	male	55	11 (20.0)	17 (30.9)	7 (12.7)	28 (50.9)	3 (5.5)	3 *B. abortus*	37, 36, 35
	female	51	4 (7.8)	7 (13.7)	8 (15.7)	5 (9.8)	6 (11.8)	6 *B. abortus*	30, 32, 33, 36, 37
*Sub-total*		106	15 (14.2)	24 (22.6)	15 (14.2)	33 (31.1)	9 (8.5)		
Aswan	male	161	30 (18.6)	41 (25.5)	39 (24.2)	51 (31.7)	7 (4.4)	6 *B. abortus*	37, 37, 36, 36, 36, 36
								1 *B. melitensis*	34
	female	25	3 (12.0)	7 (28.0)	2 (8.0)	8 (32.0)	1 (4.0)	1 *B. abortus*	36
*Sub-total*		186	33 (17.7)	48 (25.8)	41 (22.0)	59 (31.7)	8 (4.3)		
Al-Bahr Al-Ahmar (the Red Sea)	male	79	11(13.9)	13 (16.5)	20 (25.3)	24 (30.4)	14 (17.7)	8 *B. abortus*	37, 35, 31, 37, 35, 32, 36, 35
								1 *B. melitensis*	36
								5 *B. suis*	37, 36, 33, 28, 37
	female	10	0 (0.0)	2 (20.0)	1 (10.0)	2 (20.0)	1 (10.0)	1 *B. abortus*	37
*Sub-total*		89	11 (12.4)	15 (16.9)	21 (23.6)	26 (29.2)	15 (16.9)		
Grand-total		381	59 (15.5)	87 (22.8)	77 (20.2)	118 (31.0)	32 (8.4)		

* RBPT: Rose-Bengal plate test; i-ELISA: indirect ELISA; c-ELISA: competitive ELISA; CFT: complement fixation test. ** Cq/Ct-values: cycle quantification/ cycle threshold values.

**Table 3:** Relation of the risk factors with the seroprevalence and molecular detection of brucellosis in 381camels, Egypt.

Variable	Seroprevalence *n* (%)				Molecular Identification	
	RBPT *	i-ELISA *	c-ELISA *	CFT *	Real-Time PCR *n* (%)	*Brucella* DNA Identification
**Geographical location**						
Aswan (*n* = 186)	33 (17.7)	48 (25.8)	41 (22.0)	59 (31.7)	8 (4.3)	7 *B. abortus* 1 *B. melitensis*
Giza (*n* =106)	15 (14.2)	24 (22.6)	15 (14.2)	33 (31.1)	9 (8.5)	9 *B. abortus*
Al-Bahr Al-Ahmar (the Red Sea) (*n* = 89)	11 (12.4)	15 (16.9)	21 (23.6)	26 (29.2)	15 (16.9)	9 *B. abortus* 5 *B. suis* 1 *B. melitensis*
*p*-value **	0.4688	0.3205	0.4171	NA	NA	
X^2^	1.5153	2.2757	1.7489			
Df	2					
95% CI	-	-	-			
OR	-	-	-			
**Breed**						
Al-Beshary (*n* = 89)	11 (12.4)	15 (16.9)	21 (23.6)	26 (29.2)	15 (16.9)	9 *B. abortus* 5 *B. suis* 1 *B. melitensis*
Al-Ebadi (*n* = 93)	16 (17.2)	26 (28.0)	22 (23.7)	30 (32.3)	4 (4.3)	3 *B. abortus* 1 *B. melitensis*
Al-Zemkly (*n* = 106)	15 (14.2)	24 (22.6)	15 (14.2)	33 (31.1)	9 (8.5)	9 *B. abortus*
Al-Zubaidi (*n* = 93)	17 (18.3)	22 (23.7)	19 (20.4)	29 (31.2)	4 (4.3)	4 *B. abortus*
*p*-value **	0.6775	0.4658	0.5823	NA	NA	
X^2^	1.5205	2.5532	1.9524			
Df	3					
95% CI	-	-	-			
OR	-	-	-			
**Sex**						
Females (*n* = 86)	7 (8.1)	16 (18.6)	11 (12.8)	15 (17.4)	8 (9.3)	8 *B. abortus*
Males (*n* = 295)	52 (17.6)	71 (24.1)	66 (23.4)	103 (34.9)	24 (8.3)	17 *B. abortus* 5 *B. suis* 2 *B. melitensis*
*p*-value **	0.7177	0.3515	0.9164	NA	NA	
X^2^	0.13075	0.86806	0.011028			
Df	1					
95% CI	0.2819–2.2582	0.6333–3.1044	0.4215–2.4729			
OR	0.8438063	1.4091	1.0436			
**Age**						
<8 years (*n* = 227)	39 (17.2)	52 (22.9)	46 (20.2)	81 (35.7)	21 (9.3)	15 *B. abortus* 5 *B. suis* 1 *B. melitensis*
≥8–11 years (*n* = 68)	13 (19.1)	19 (27.9)	20 (29.4)	22 (32.4)	3 (4.4)	2 *B. abortus* 1 *B. melitensis*
11–13 years (*n* = 51)	4 (7.8)	7 (13.7)	8 (15.7)	5 (9.8)	6 (11.8)	6 *B. abortus*
>13–15 years (*n* = 35)	3 (8.6)	9 (25.7)	3 (8.6)	10 (28.6)	2 (5.7)	2 *B. abortus*
*p*-value **	0.7844	0.5792	0.1672	NA	NA	
X^2^	1.0699	1.9674	5.0641			
Df	3					
95% CI	-	-	-			
OR	-	-	-			

* The univariate analysis was based on the RBPT, i-ELISA and c-ELISA results; OR: odds ratio; CI: confidence interval; df: degree of freedom; X^2^: Pearson’s Chi-squared test; Ref: reference. ** (Statistical value of significance: *p*-value ≤ 0.05).

## References

[1] Kirk M.D., Pires S.M., Black R.E., Caipo M., Crump J.A., Devleesschauwer B., Döpfer D., Fazil A., Fischer-Walker C.L., Hald T. (2015). World Health Organization Estimates of the Global and Regional Disease Burden of 22 Foodborne Bacterial, Protozoal, and Viral Diseases, 2010: A Data Synthesis. PLoS Med..

[2] Fatima S., Khan I., Nasir A., Younus M., Saqib M., Melzer F., Neubauer H., El-Adawy H. (2016). Serological, molecular detection and potential risk factors associated with camel brucellosis in Pakistan. Trop. Anim. Health Prod..

[3] Gyuranecz M., Wernery U., Kreizinger Z., Juhász J., Felde O., Nagy P. (2016). Genotyping of Brucella melitensis strains from dromedary camels (*Camelus dromedarius*) from the United Arab Emirates with multiple-locus variable-number tandem repeat analysis. Veter Microbiol..

[4] Gwida M., Elgohary A., Melzer F., Khan I., Rösler U., Neubauer H. (2012). Brucellosis in camels. Res. Veter Sci..

[5] Sayed-Ahmed M., Mohamed Z.S.-A., Mohamed M.E.-D., Mohamed A.E.-B., Sherif M.S., Emad E.Y., El-Sayed A.M., El-Diasty M.M., El-Beskawy M.A., Shoieb S.M. (2017). Sero-prevalence of camel brucellosis (*Camelus dromedarius*) and phenotypic characteristics of Brucella melitensis biovar 3 in Shalateen City, Red Sea Governorate, Egypt. Afr. J. Microbiol. Res..

[6] Salisu U., Kudi C., Bale J., Babashani M., Kaltungo B., Saidu S., Asambe A., Baba A. (2017). Seroprevalence of *Brucella* antibodies in camels in Katsina State, Nigeria. Trop. Anim. Health Prod..

[7] Sprague L.D., al Dahouk S., Neubauer H. (2012). A review on camel brucellosis: A zoonosis sustained by ignorance and indifference. Pathog. Glob. Health.

[8] Othman O.E., El-Kader H.A.A., Alam S.S., El-Aziem S.H.A. (2017). Cytochrome b conservation between six camel breeds reared in Egypt. J. Genet. Eng. Biotechnol..

[9] Refai M. (2002). Incidence and control of brucellosis in the Near East region. Veter Microbiol..

[10] Menshawy A., Perez-Sancho M., García-Seco T., Hosein H.I., García N., Martínez I., Sayour A., Goyache J., Azzam R.A.A., Domínguez L. (2014). Assessment of Genetic Diversity of Zoonotic *Brucella* spp. Recovered from Livestock in Egypt Using Multiple Locus VNTR Analysis. BioMed Res. Int..

[11] Wareth G., Hikal A., Refai M., Melzer F., Roesler U., Neubauer H. (2014). Animal brucellosis in Egypt. J. Infect. Dev. Ctries..

[12] Hegazy Y.M., Molina-Flores B., Shafik H., Ridler A., Guitian F., Guitian J. (2011). Ruminant brucellosis in Upper Egypt (2005–2008). Prev. Veter Med..

[13] Eltholth M.M., Hegazy Y.M., El-Tras W.F., Rushton J., Bruce M. (2016). Temporal Analysis and Costs of Ruminant Brucellosis Control Programme in Egypt Between 1999 and 2011. Transbound. Emerg. Dis..

[14] Samaha H., Al-Rowaily M., Khoudair R.M., Ashour H.M. (2008). Multicenter Study of Brucellosis in Egypt. Emerg. Infect. Dis..

[15] Khan A.U., Melzer F., El-Soally S.A.G.E., Elschner M.C., Mohamed S.A., Ahmed M.A.S., Roesler U., Neubauer H., El-Adawy H. (2019). Serological and Molecular Identification of *Brucella* spp. in Pigs from Cairo and Giza Governorates, Egypt. Pathogens.

[16] Nielsen K., Yu W.L. (2010). Serological diagnosis of brucellosis. Prilozi.

[17] Mathew C., Stokstad M., Johansen T.B., Klevar S., Mdegela R.H., Mwamengele G., Michel P., Escobar L., Fretin D., Godfroid J. (2015). First isolation, identification, phenotypic and genotypic characterization of *Brucella abortus* biovar 3 from dairy cattle in Tanzania. BMC Veter Res..

[18] Ulu-Kilic A., Metan G., Alp E. (2013). Clinical presentations, and diagnosis of brucellosis. Recent Pat. Antiinfect Drug Dis..

[19] Gwida M., Elgohary A., Melzer F., Tomaso H., Roesler U., Wernery U., Wernery R., Elschner M., Khan I., Eickhoff M. (2011). Comparison of diagnostic tests for the detection of *Brucella* spp. in camel sera. BMC Res. Notes.

[20] Garcell H.G., Garcia E.G., Pueyo P.V., Martín I.R., Arias A.V., Serrano R.N.A. (2016). Outbreaks of brucellosis related to the consumption of unpasteurized camel milk. J. Infect. Public Health.

[21] Rhodes H.M., Williams D.N., Hansen G.T. (2016). Invasive human brucellosis infection in travelers to and immigrants from the Horn of Africa related to the consumption of raw camel milk. Travel Med. Infect. Dis..

[22] Ben-Shimol S., Dukhan L., Belmaker I., Bardenstein S., Sibirsky D., Barrett C., Greenberg D. (2012). Human brucellosis outbreak acquired through camel milk ingestion in southern Israel. Isr. Med Assoc. J. IMAJ.

[23] Zakaria A.M., Ahmed S.F., Motawae M.S. (2018). Seropositivity in animals and risk of occupational brucellosis among abattoirs personnel associated with poor work practices and absence of safety policy in Egypt. Int. J. Occup. Environ. Health.

[24] OIE (2019). Brucellosis (*Brucella abortus, B. melitensis* and *B. suis*) (infection with *B. abortus, B. melitensis* and *B. suis*). Manual of Diagnostic Tests and Vaccines for Terrestrial Animals 2019, OIE.

[25] Probert W.S., Schrader K.N., Khuong N.Y., Bystrom S.L., Graves M.H. (2004). Real-Time Multiplex PCR Assay for Detection of *Brucella* spp., *B. abortus*, and *B. melitensis*. J. Clin. Microbiol..

[26] Hänsel C., Mertens K., Elschner M.C., Melzer F. (2015). Novel real-time PCR detection assay for *Brucella suis*. Veter Rec. Open.

[27] Sanogo M., Abatih E., Thys E., Fretin D., Berkvens D., Saegerman C. (2013). Importance of identification and typing of *Brucellae* from West African cattle: A review. Vet. Microbiol..

[28] Godfroid J., al Dahouk S., Pappas G., Roth F., Matope G., Muma J., Marcotty T., Pfeiffer D.U., Skjerve E. (2013). A “One Health” surveillance and control of brucellosis in developing countries: Moving away from improvisation. Comp. Immunol. Microbiol. Infect. Dis..

[29] Machavarapu M., Poonati R., Mallepaddi P.C., Gundlamadugu V., Raghavendra S., Polavarapu K.K.B., Polavarapu R. (2019). Endemic brucellosis in Indian animal and human populations: A billion-dollar issue. J. Curr. Trends Biotechnol. Pharm..

[30] Godfroid J. (2017). Brucellosis in livestock and wildlife: Zoonotic diseases without pandemic potential in need of innovative one health approaches. Arch. Public Health.

[31] Musa M., Eisa M., el Sanousi E., Wahab M.A., Perrett L. (2008). Brucellosis in Camels (*Camelus dromedarius*) in Darfur, Western Sudan. J. Comp. Pathol..

[32] Chisholm K., Dueger E., Fahmy N.T., Samaha H.A.T., Zayed A., Abdel-Dayem M.S., Villinski J.T. (2012). Crimean-Congo Hemorrhagic Fever Virus in Ticks from Imported Livestock, Egypt. Emerg. Infect. Dis..

[33] Wakene W.Z. (2017). Review on Epidemiology of Camel and Human Brucellosis in East Africa, Igad Member Countries. Sci. J. Clin. Med..

[34] Ibrahim H.H., Rouby S., Menshawy A., Ghazy N. (2016). Seroprevalence of Camel Brucellosis and Molecular Characterization of *Brucella melitensis* Recovered from Dromedary Camels in Egypt. Res. J. Vet. Pr..

[35] Moghney A.R.F.A. (2004). A preliminary study on brucellosis on camels at Behira Province. Ass. Univ. Bull. Environ. Res..

[36] Sayour A.E., Elbauomy E., Abdel-Hamid N.H., Mahrous A., Carychao D., Cooley M.B., Elhadidy M. (2020). MLVA fingerprinting of *Brucella melitensis* circulating among livestock and cases of sporadic human illness in Egypt. Transbound. Emerg. Dis..

[37] Hamdy M., Amin A. (2002). Detection of *Brucella* Species in the Milk of Infected Cattle, Sheep, Goats and Camels by PCR. Vet. J..

[38] Abdel-Hamid N.H., Elbauomy E.M., Ghobashy H.M., Sayour A.E., Ismail R.I., Soliman H.S., Abdel-Haleem M.H. (2017). Role of sheep and goat mobile flocks in the transmission of brucellosis to the household ruminants and the disease prevalence in these flocks. Anim. Health Res. J..

[39] Omer M., Musa M., Bakhiet M., Perrett L. (2010). Brucellosis in camels, cattle, and humans: Associations and evaluation of serological tests used for diagnosis of the disease in certain nomadic localities in Sudan. Rev. Sci. Tech. OIE.

[40] Musa M., Jahans K., Fadalla M. (1990). *Brucella* Biovars isolated from nomadic cattle in the Southern Darfur Province of Western Sudan. J. Comp. Pathol..

[41] Gumaa M., Osman H., Omer M., el Sanousi E., Godfroid J., Ahmed A. (2014). Seroprevalence of brucellosis in sheep and isolation of *Brucella abortus* biovar 6 in Kassala State, Eastern Sudan. Rev. Sci. Tech. OIE.

[42] Agab H., Abbas B., el Jack Ahmed H., Maoun I.E. (1994). First report on the isolation of *Brucella abortus* biovar 3 from camel (*Camelus dromedarius*) in the Sudan. Rev. Elev. Med. Vet. Pays. Trop..

[43] Faye B., Abdelhadi O.M.A., Ahmed A.I., Bakheit S.A. (2011). Camel in Sudan: Prospects. Livestock Res. Rural Develop..

[44] Ibrahim S.I. (1996). Studies on swine brucellosis in Egypt. J. Egypt Vet. Med. Ass..

[45] Gilbert M., Nicolas G., Cinardi G., van Boeckel T.P., Vanwambeke S.O., Wint G.R.W., Robinson T.P. (2018). Global distribution data for cattle, buffaloes, horses, sheep, goats, pigs, chickens, and ducks in 2010. Sci. Data.

[46] Coelho A., Díez J.G., Coelho A.M. (2015). Risk Factors for *Brucella* spp. in Domestic and Wild Animals. Updates on Brucellosis.

[47] Abebe G., Worku Y., Mamo G., Nazir S. (2017). Sero-prevalence and Associated Risk Factors of Brucellosis in Camel at Akaki Abattoir, Central Ethiopia. J. Anim. Res..

[48] Ullah S. (2015). Prevalence of Brucellosis among Camels in District Muzaffargarh, Pakistan. J. Infect. Mol. Boil..

[49] Bayasgalan C., Chultemdorj T., Roth F., Zinsstag J., Hattendorf J., Badmaa B., Argamjav B., Schelling E. (2018). Risk factors of brucellosis seropositivity in Bactrian camels of Mongolia. BMC Veter Res..

[50] Al-Majali A.M., Al-Qudah K., Al-Tarazi Y.H., Al-Rawashdeh O.F. (2007). Risk factors associated with camel brucellosis in Jordan. Trop. Anim. Health Prod..

[51] Wernery U. (2014). Camelid brucellosis: A review. Rev. Sci. Tech. OIE.

[52] Sayour A.E., Elbauomy E.M., Shehata A.A., El-Kholi M.K. (2015). Brucellosis prevalence and serological profile of male one-humped camels reared in Somaliland and eastern Ethiopia for meat production. Global Vet..

